# Subcritical Water as Pretreatment Technique for Bioethanol Production from Brewer’s Spent Grain within a Biorefinery Concept

**DOI:** 10.3390/polym14235218

**Published:** 2022-11-30

**Authors:** Patricia Alonso-Riaño, Mariana S. T. Amândio, Ana M. R. B. Xavier, Sagrario Beltrán, Maria Teresa Sanz

**Affiliations:** 1 Department of Biotechnology and Food Science, University of Burgos, Plaza Misael Bañuelos s/n, 09001 Burgos, Spain; 2 Chemistry Department, CICECO—Aveiro Institute of Materials, University of Aveiro, 3810-193 Aveiro, Portugal; 3 Department of Chemical Engineering CIEPQPF, Faculty of Sciences and Technology, University of Coimbra, 3030-790 Coimbra, Portugal

**Keywords:** subcritical water pretreatment, brewer’s spent grain, lignocellulosic biomass, bioethanol, high solids’ loading, simultaneous saccharification and fermentation, separate hydrolysis and fermentation

## Abstract

Bioeconomy and environmental issues envisage industrial by-products such as Brewer’s spent grain (BSG) as renewable resources for their recycling and reuse within a biorefinery concept. This study aimed to investigate the production of bioethanol from subcritical water (subW) pretreated BSG, following the conversion of the BSG biopolymers cellulose and hemicelluloses. The subW pretreatment was performed in a batch reactor at 174 °C, during 60 min and 5% (*w/v*) of dry BSG charge. The behavior of BSG biopolymers under subW pretreatment was monitored by evaluating the chemical composition of the liquid and solid streams and the chemical and structural changes caused in the solid residues by scanning electron microscope (SEM), CHNS elemental analysis and water retention value (WRV). The production of bioethanol from subW-pretreated BSG was assessed by separate hydrolysis and fermentation (SHF) and also by simultaneous saccharification and fermentation (SSF) by using the enzymatic cocktail Celluclast 1.5 L (40 FPU/g_solids_) and the yeast Ethanol Red^®^. The higher bioethanol productivity (1.073 g∙L^−1^∙h^−1^) and concentration (32.18 g/L) were achieved by SSF with higher solids’ loading (25%) and following a fed-batch strategy. These results suggest that subcritical water pretreatment is a promising technology for the valorization of BSG as a feedstock for second-generation bioethanol production.

## 1. Introduction

Biorefinery has been considered a promising concept for processing biomass into different products and energy, being very important in the context of circular economy, closing loops of streams, and valorizing multiple outputs [1]. In this regard, Sganzerla et al. [2] demonstrated the possibility of developing a biorefinery using brewer’s spent grain (BSG) as raw material to obtain several valuable compounds such as arabinoxylans, proteins, ferulic acid, xylitol, xylose, lactic acid, butanol, biogas, fertilizer, and bioethanol. 

BSG is the main solid by-product generated in breweries, which remains after the mashing and wort filtration process, representing 85% of the total by-products generated. It was estimated that about 20 kg of BSG are produced per 100 L of beer [3]. BSG is a lignocellulosic material mainly composed of hemicelluloses, cellulose, protein, and lignin, the polysaccharides fraction being more than 50% of the BSG composition on a dry weight basis [4]. The monomeric sugars required to feed the fermentation processes to produce biofuels can be released by enzymatic saccharification of the biopolymers’ cellulose and hemicelluloses. However, a pretreatment step is needed before performing enzymatic saccharification due to the complex morphological macrostructure of lignocellulosic materials and their characteristic recalcitrance [5,6]. Different pretreatment methods have been applied, acid and alkaline treatments being the most commonly used [7].

The use of subcritical water (subW) as an eco-friendly method for lignocellulose pretreatment avoiding chemicals addition was proposed in recent studies [8,9,10,11]. SubW is pressurized water in its liquid state in the temperature range from 100 °C to 374 °C. Under these conditions, water presents unique properties such as higher ionic product and lower dielectric constant than at ambient conditions [12]. The hydrogen bonds of subW gradually weaken with temperature, producing more acidic hydronium ions (H_3_O^+^) and alkaline hydroxide ions (OH^–^), promoting hydrolysis reactions and the catalytic effect of hydronium ions [12]. Hydrothermal extraction at mild conditions (160–210 °C) is a promising technique for hemicelluloses hydrolysis [12]. During the subW pretreatment, the hemicelluloses fraction is mostly hydrolyzed/solubilized. This way, the sugars present in the hemicelluloses fraction are released (as oligomers and/or monomers), and the remaining solid is enriched in cellulose and lignin [8,11,13]. In addition, during subW pretreatment, different bioactive compounds can be released from the biomass, such as phenolic compounds, protein, and amino acids [7]. The recovery of these valuable compounds during the pretreatment, as well as fermentable carbohydrates, constitutes a significant advance that could enhance the feasibility of the industrial implementation of lignocellulosic biomass-based biorefineries [1].

The cellulose-rich fraction remaining after the subW pretreatment can be subjected to enzymatic hydrolysis. This biopolymeric cellulosic fraction can feed bioethanol production [11], and different fermentation strategies can be employed, namely separate hydrolysis and fermentation (SHF) and simultaneous saccharification and fermentation (SSF). The main advantage of SHF is that the hydrolysis and the fermentation processes can be performed under their different optimal conditions, around 50 °C for the enzymatic hydrolysis and 28–30 °C for the yeast fermentation. However, using SSF instead of SHF may reduce the total time required for bioethanol production from BSG increasing productivity [14]. Furthermore, the use of high solids’ loading may increase bioethanol concentration and, consequently, reduce production costs of the lignocellulose-to-bioethanol process [8].

The main novelty of this work is the valorization of brewer’s spent grain within a biorefinery concept using environmentally friendly subcritical water and avoiding chemicals’ addition, focusing on cellulosic bioethanol production. The effectiveness of subW as pretreatment to improve the BSG enzymatic hydrolysis yield of glucose by using cellulases at different solid loadings was evaluated. A comparison of different fermentation configurations, such as SHF and SSF, was performed, namely in terms of bioethanol concentration and productivity. Furthermore, one of the main goals of this work was to improve bioethanol production from BSG by increasing solids’ loading up to 25% by following a fed-batch strategy and reducing overall time when performing SSF. In addition, the overall process overview for bioethanol production from BSG, under the different conditions, was presented and discussed to valorize all fractions obtained along the process, in the context of a biorefinery.

## 2. Materials and Methods

### 2.1. Raw Material

The raw material used in this work was the brewer’s spent grain kindly supplied by San Miguel S.A, from the industrial brewery located in Burgos (Spain). This raw material was first preconditioned, as soon as obtained, by washing it with water until uncolored washing water was observed and drying it in an air convection oven (45 °C) until reaching a final moisture content of 8% (*w/w*).

The dry BSG was milled in a SM100 mill (Retsch, Haan, Germany) to obtain a particle size lower than 0.5 mm. Biomass characterization was performed according to the NREL protocols [15]. Carbohydrates were quantified by high-performance liquid chromatography (HPLC) with a Bio-Rad Aminex-HPX-87 H column (Hercules, CA, USA) and an Agilent Technologies (Waldbronn, Germany) refractive index detector (RID), maintained at 40 °C. The mobile phase was 0.005 M sulfuric acid from Merck (Darmstadt, Germany). Megazyme Total Starch Assay and β-Glucan Assay (Wicklow, Ireland) were followed to determine starch and β-glucans content in the BSG. Protein in the raw material was estimated from the nitrogen content present in the samples as measured by the elemental analysis and considering a nitrogen factor of 6.25 according to the amino acid profile of the protein fraction of the BSG [7].

### 2.2. Subcritical Water Hydrolysis

Subcritical water hydrolysis treatment was carried out in a discontinuous stainless-steel reactor of 0.5 L maximum capacity. The heating system consisted of a heating jacket of 230 V and 400 W covering the reactor, used to reach the working temperature. A Pt100 sensor connected to a PID system and placed inside the reactor helps to control and register the temperature during the hydrolysis. The biomass was loaded into the reactor and filled with water. The mixture was heated up to the desired temperature and pressure was fixed at 50 bars using nitrogen gas and maintained during the process. SubW hydrolysis was carried out at 174 ± 3 °C for a total treatment time of 60 min and a biomass loading of 5% (*w/v*).

The liquid stream was analyzed in terms of protein content, monomeric sugars, and oligomeric sugars. Monosaccharides were determined by HPLC, as described in the Section 2.1, whereas total sugars (monomeric + oligomeric sugars) were determined after a first acid hydrolysis step in order to release monomeric sugars from oligomers for quantification following the NRLE protocols [16]. Protein content in the liquid stream was determined from the nitrogen content measured by using a TOC/TN analyzer (Shimadzu TOC-V CSN analyzer, Tokio, Japan) using KNO_3_ as a standard after applying the same nitrogen factor as that used for the BSG protein fraction, 6.25.

The remaining solid after subW treatment (pretreated BSG) was washed to remove the residual sugars and inhibitors before drying it at 45 °C. Chemical characterization of pretreated BSG was performed as described for the raw material. The washing water was analyzed to ensure no residual compounds remained on the solid residue. Several subW batches were performed at the same conditions in the 0.5 L reactor, the remaining solids were collected, and the mix of all of them was used as the pretreated BSG for further enzymatic hydrolysis. 

### 2.3. Enzymatic Hydrolysis

A cellulolytic cocktail (Celluclast 1.5 L) kindly provided by Novozymes A/S (Bagsværd, Denmark) was utilized to hydrolyze both raw BSG and pretreated BSG. Celluclast 1.5 L is a liquid cellulases cocktail with 1.2 g/mL of density and declared activity of 700 endo-glucanase units/g. Its filter paper unit (FPU) activity was determined according to the NREL standard procedure [17] as 53.7 FPU/mL. The enzymatic loading was always 40 FPU/g of solid in the presence of a 0.05 M sodium citrate buffer at pH 4.8. The effect of solids’ loading in the enzymatic hydrolysis yield was studied at 5, 10, 15 and 20% (*w/v*) for untreated BSG and at 5 and 8% (*w/v*) for subW-pretreated BSG, based on oven-dried material. The experiments were conducted for 94 h, with a stirring rate of 100 rpm, at 50 °C and samples were taken at 12, 22, 46, 70, and 94 h. The reaction was stopped by heating the samples in boiling water for 5 min for enzymes’ denaturation. After cooling the samples in ice for 5 min, they were centrifuged at 13,000 rpm for 10 min. After proper dilution, the supernatant was analyzed for monomeric sugars and lactic acid determination. Furthermore, blanks of the enzymatic solutions were prepared and analyzed by HPLC, to subtract the sugar content since the commercial cellulolytic complex enzymes could contain some sugar. These assays were performed in duplicate.

### 2.4. Microorganism

*Saccharomyces cerevisiae* (Ethanol Red^®^) was kindly provided by Leaf by Lesaffre Advanced Fermentations (Marcq-en-Baroeul, France). This strain was grown at 28 °C and maintained at 4 °C on Petri dishes with solid yeast medium (YM) prepared with 10 g/L glucose, 5 g/L peptone, 3 g/L malt extract and 3 g/L yeast extract (the pH adjusted to 5.5), and 20 g/L agar.

### 2.5. Pre-Inoculum and Inoculum

Pre-inoculum was prepared by transferring a colony from a maintenance YM Petri dish to 10 mL liquid YM (similar to solid YM, except agar) and was incubated for 24 h at 28 °C and 180 rpm. The inoculum was prepared by transferring the pre-inoculum to 40 mL of fresh liquid YM. The inoculum was incubated at 28 °C and 180 rpm for 14 h. These procedures were carried out in duplicate. 

### 2.6. Fermentation

**Figure 1:**
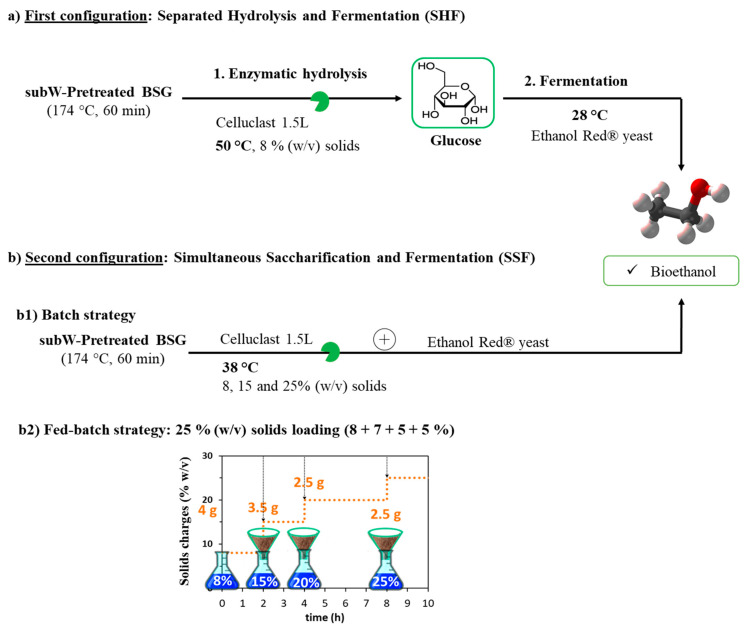
Hydrolysis and fermentation configurations employed in this work. (**a**) Separate hydrolysis and fermentation (SHF) at 8% (*w/v*) solids loading. (**b**) Simultaneous saccharification and fermentation (SSF) followed in (**b1**) batch (8, 15, and 25% *w/v* solids loading) or in (**b2**) fed-batch operation mode to reach a high solids’ loading of 25% (*w/v*).

Different saccharification and fermentation strategies have been followed to obtain bioethanol from the subcritical water-pretreated BSG. These strategies have been represented in Figure 1. 

#### 2.6.1. Separate Hydrolysis and Fermentation Assays (SHF)—1st Configuration

The hydrolysate obtained after 48 h of enzymatic hydrolysis of the subW-pretreated BSG at 8% (*w*/*v*) of solids loading (see Section 2.3) was centrifuged for 1 h at 5000 rpm and 4 °C (Megafuge 16R, Thermo Scientific, Osterode am Harz, Germany). The supernatant was sterilized by autoclaving at 121 °C for 20 min (Uniclave 88, AJC, Cacém, Portugal). The resulting hydrolysate was analyzed by HPLC and submitted to fermentations in 250 mL Erlenmeyer flasks with a working volume of 50 mL, incubated at 28 °C and 180 rpm. Supplementation was composed by 2.0 g/L (NH_4_)_2_HPO_4_, 1.0 g/L (NH_4_)_2_SO_4_, 0.5 g/L MgSO_4_·7H_2_O, and 2.5 g/L yeast extract. Fermentation media was composed of 85% (*v/v*) hydrolysate, 5% (*v/v*) of supplementation solution, the exact volume of inoculum that guarantees an optical density at 620 nm (OD620 nm) of about 0.400, and NaCl solution (0.9%) to reach the final volume. Sterile samples were collected periodically, and after biomass and pH monitorisation, they were centrifuged at 13,000 rpm for 10 min. The supernatant was kept to determine glucose, xylose, glycerol, and ethanol concentrations.

#### 2.6.2. Simultaneous Saccharification and Fermentation (SSF)—2nd Configuration

The SSF experiments were performed in 250 mL Erlenmeyer flasks with a total volume of 50 mL of citrate buffer (0.05 M, pH 4.8) containing the nutrients previously described for SHF and the exact volume of inoculum that guarantees an OD_620 nm_ of about 0.400.

#### Batch Strategy

The substrates were subW-pretreated BSG at 8%, 15% and 25% (*w/v*) solids loading based on oven-dried material. SSF was started by adding the enzymatic consortium and inoculum simultaneously. Enzyme dosages used were the same as in the enzymatic hydrolysis experiments. The assays were carried out at 38 °C and 180 rpm. Sampling was performed as described in Section 2.6.1.

#### Fed-Batch Strategy

A fed-batch approach was followed with an initial solids’ loading of 4 g, 8% (*w/v*) based on the total working volume (50 mL), where 3.5 g of subW-pretreated BSG were fed after 2 h and more two doses of 2.5 g were added after 4 h and 8 h from the beginning, achieving a total concentration of 25% (*w/v*) (see Figure 1b). The assays were carried out at 38 °C and 180 rpm in a total working volume of 50 mL. Sampling was performed as described in Section 2.6.1.

### 2.7. Analytical Methods

The pH was measured using an electrode InPro 3030/200 (Mettler Toledo, Columbus, OH, USA) connected to a benchtop meter sensION+ MM340 (Hach, Loveland, CO, USA).

Biomass was monitored by measuring optical density at 620 nm (UVmini-1240, Shimadzu, Tokyo, Japan) and further converted into concentration using a calibration curve of optical density versus biomass dry weight.

High-performance liquid chromatography (HPLC) was used to quantify glucose, xylose, arabinose, lactic acid, glycerol, and ethanol. After being centrifuged and filtered for 8 min at 8000 rpm (Eppendorf, Hamburg, Germany), samples were injected by autosampler L-2200 (MiniSprin centrifuge, Hitachi, Ltd., Chiyoda, Japan) on a Rezex ROA-Organic Acid H+ (8%) 300 × 7.8 mm ion-exchange column (Phenomenex, Torrance, CA, USA) at 65 °C (oven Gecko 2000, CIL Cluzeau, Sainte-Foy-la-Grande, France) and detected by a refraction index detector L-2490 (Hitachi, Chiyoda, Japan). The injection volume was 10 µL and the eluent used was H_2_SO_4_ 0.005 N, with a flow rate of 0.5 mL/min (pump L-2130, Hitachi). A standard calibration curve was used for the determination of metabolites’ concentration.

The Water Retention Value (WRV) of untreated BSG and subW-pretreated BSG were determined following the method described in Lv et al. [18], with some modifications. Firstly, m_0_ = 0.5 g of substrate (dry weight) was placed into a centrifugal tube with 5 mL of deionized water for 2 h. The solid and liquid phases were separated by centrifugation at 3000 g for 15 min and weighed. The wet solid was weighed (m_1_), oven dried at 105 °C until constant weight, and weighed again (m_2_) to determine the mass of water evaporated (m_w-e_). Two aliquots of supernatant (1 g) were weighed, filtered, oven dried at 105 °C until constant weight, and it was weighed again to determine the mass fraction of soluble solids (y_ss_) and, by difference, the mass fraction of water (y_w_). Assuming that the solution adhered to the solids has the same composition as the supernatant [19], the mass of soluble solids (m_ss_) in the solid phase was calculated according to Equation (1).

(1) $$m_{\mathrm{ss}}=m_{w-e}\frac{y_{ss}}{y_{w}}$$

The mass of insoluble solids (m_is_) was calculated according to Equation (2)

(2) $$m_{\mathrm{is}}=m_{2}-m_{w-e}-m_{\mathrm{ss}}$$

The WRV was expressed as the percentage of water retained by mass of insoluble solids, according to the Equation (3):

(3) $$\mathrm{WRV}\;\left(\%\right)=\frac{m_{w-e}}{m_{\mathrm{is}}}\cdot 100$$

Scanning electron micrographs (SEM) of raw BSG and subW-pretreated BSG were taken by a Scanning Electron Microscope JEOL JSM-6460LV with Energy Dispersive X-ray (JEOL Ltd. Tokio, Japan) operating at 20 kV, at different magnifications, after gold-sputtering samples.

Elemental composition (C, H, N, S, O) of raw BSG, subW-pretreated BSG and the solid residues remained after SHF (8% solids loading) and SSF (25% solids loading) were determined. The high heating value (HHV) of the solids was evaluated by Equation (4) proposed by Friedl et al. [20].

(4) $$\mathrm{HHV}\;\left(\mathrm{kJ}/\mathrm{kg}\right)=3.55C^{2}-232C-2230H+51.2C\cdot H+131N+20600$$

All analytical determinations were performed in triplicate. Average results, with standard deviations lower than 5%, were reported.

### 2.8. Calculations

The glucose and xylose yields in the enzymatic hydrolysis were evaluated as the ratio of the amount of sugar released to the medium over the maximum potential yield of sugar obtained from the solid (raw BSG and subW-pretreated BSG) characterization, according to Equation (5):

(5) $${\mathrm{Yield}}_{\mathrm{sugar}}\;\left(\%\right)=\frac{\mathrm{Monomeric}\;\mathrm{sugar}\;\mathrm{in}\;\mathrm{the}\;\mathrm{enzymatic}\;\mathrm{hydrolysate}}{\mathrm{Sugar}\;\mathrm{in}\;\mathrm{solid}\;\mathrm{BSG}}$$

The ethanol yield in SHF was calculated according to Equation (6) to compare the results with values reported in the literature.

(6) $$\mathrm{Ethanol}\;\mathrm{yield},\;\mathrm{SHF}\;\left(\%\right)=\frac{\mathrm{Ethanol}\;\mathrm{produced}\;}{{\mathrm{Glucose}}_{\mathrm{solution}}\times 0.511\;}\cdot 100$$

Ethanol yield in SSF was calculated according to Equation (7). The ethanol yield in SHF was also calculated according to this equation, considering the dilution during the fermentation media preparation (Section 2.6.1), to compare the results with the SSF results on the same basis.

(7) $$\mathrm{Ethanol}\;\mathrm{yield},\;\mathrm{SSF}\;\left(\%\right)=\frac{\mathrm{Ethanol}\;\mathrm{produced}\;}{{\mathrm{Glucan}}_{\mathrm{solid}}\times 1.111\times 0.511\;}\cdot 100$$

where 1.111 (180/162) is the stoichiometric factor for glucan hydration from glucose and 0.511 is the glucose conversion into ethanol considering the maximal theoretical yield of ethanol (2 mole of ethanol produced from 1 mole of glucose).

The volumetric ethanol productivity, Prod_vol_ (g·L^−1^·h^−1^) was calculated, considering the time when the maximum ethanol concentration was achieved:

(8) $${\mathrm{Prod}}_{\mathrm{vol}}\left(g\cdot {\;L}^{-1}h^{-1}\right)=\frac{{\left[\mathrm{ethanol}\right]}_{\max}}{\Delta t}$$

The ethanol yield coefficient per glucose, Y_ethanol/glucose_ (g/g) and the biomass yield coefficient, Y_biomass/substrate_ (g/g), were calculated according to Equations (9) and (10), respectively, considering glucose and xylose as substrates to cell growth.

(9) $$Y_{\mathrm{ethanol}/\mathrm{glucose}}=-\frac{\Delta \left[\mathrm{ethanol}\right]}{\Delta \left[\mathrm{glucose}\right]}$$

(10) $$Y_{\mathrm{biomass}/\mathrm{substrate}}=-\frac{\Delta \left[\mathrm{biomass}\right]}{\Delta \left[\mathrm{substrate}\right]}$$

### 2.9. Statistical Analysis

All values were expressed as mean ± standard deviation from triplicate measurements. The significance of the differences was determined based on an analysis of the variance with the Fisher’s Least Significant Difference (LSD) method at *p*-value ≤ 0.05 using the Statgraphics Centurion 19 X64 software (The Plains, VA, United States). Error bars in all graphs are 95% confidence intervals.

## 3. Results and Discussion

### 3.1. Subcritical Water Pretreatment

The chemical composition of the BSG employed in this work is presented in Table 1, together with the chemical composition of pretreated BSG under subcritical water conditions on a weight percentage dry basis. The amount of carbohydrates (glucan, xylan and arabinan) present as oligomers and monomers, and the protein content in the subW hydrolysates per 100 g of dry raw BSG are also collected in Table 1. 

**Figure 2:**
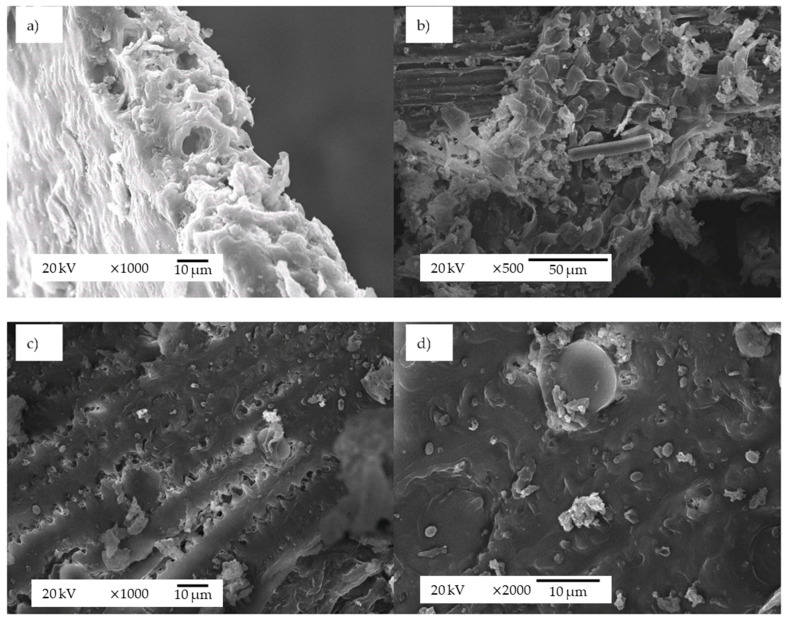
Scanning electron micrographs (SEM) of (**a**) raw BSG (1000×), (**b**) subW-pretreated BSG (500×), (**c**) subW-pretreated BSG (1000×), and (**d**) subW-pretreated BSG (2000×).

After subW treatment, 41.2% of solids remained into the reactor. Total protein content decreased from 22.1 ± 0.7% (*w/w*) in raw BSG to 12.1 ± 0.1% (*w/w*) in subW-pretreated BSG, due to the solubilization of 67% of protein content in raw BSG during the treatment. The main polysaccharide fraction of raw BSG was hemicelluloses, with 32.0 ± 0.6% (*w/w*), and the cellulose fraction was 14.0 ± 0.2% (*w/w*). In contrast, the subW-pretreated BSG demonstrated a 1.8-fold higher glucans content than raw BSG. Furthermore, most of the hemicelluloses’ fraction was removed from the BSG and recovered in the subW hydrolysates. Arabinose was not detected in the subW-pretreated BSG, according to our analytical methodology. The cellulose/hemicelluloses ratio in raw BSG was 0.44, while in the subW residue, this ratio significantly increased up to 15. On the other hand, the lignin content was 2.3-fold higher in the pretreated BSG due to the extraction of other compounds that were more easily hydrolyzed. The presence of lignin has been considered a factor that largely determines the very low enzymatic hydrolysis extension of untreated lignocellulosic biomasses, as it hinders the accessibility of enzymes to cellulose [21]. On the other hand, the insoluble lignin-soluble lignin ratio is a parameter indicating the effects of heat treatment in BSG [9]. This ratio was 3.5-fold higher in the residue obtained after subcritical water treatment than in raw BSG, suggesting that the hydrothermal treatment has caused changes in the chemical structure of lignin. The structural morphology of the samples before and after the subW pretreatment can be observed in Figure 2. Raw BSG presents a rigid and uniform structure (Figure 2a), whereas the subW-pretreated BSG exhibits separated fibers from the initial connected structure (Figure 2b), and pores on the surface (Figure 2c) that can be related to the hemicelluloses’ removal during the subW treatment [18]. Furthermore, the surface of the subW-pretreated BSG shows lignin droplets, as can be observed in Figure 2d. Hydrothermal treatment may cause condensation reactions and structural alteration in the lignin [22]. The migration and relocation of lignin could cause structural changes, resulting in an increase in pore volume and specific surface area, which facilitates enzymatic hydrolysis [21], although a negative effect of this phenomenon on enzymatic digestibility has also been hypothesized [23].

The WRV of the raw BSG was 405 ± 10% and it increased after the subcritical water pretreatment until 527 ± 4%. The increase in the WRV can be attributed to the xylan removal during the pretreatment [18]. All these chemical and structural changes in the pretreated solid can facilitate the enzymatic hydrolysis of subW-pretreated lignocellulose [24].

### 3.2. Enzymatic Hydrolysis of Untreated BSG and Subcritical Water-Pretreated BSG

#### 3.2.1. Untreated BSG

**Figure 3:**
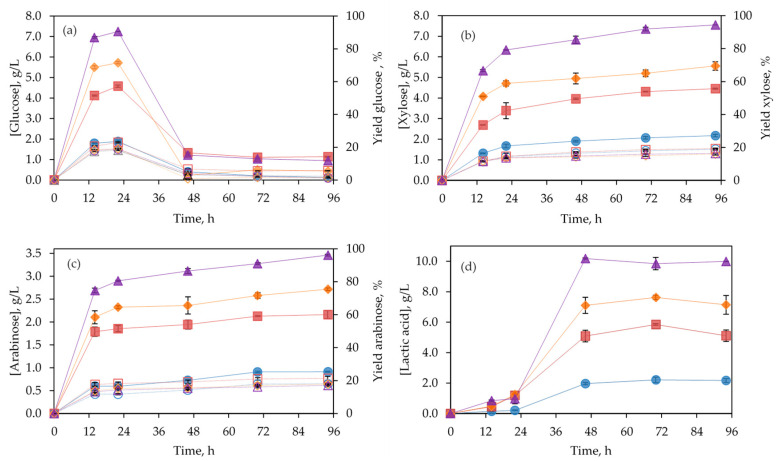
Time course of enzymatic hydrolysis of raw BSG at different solids’ loading ●○5% (*w/v*), ■□ 10% (*v/w*), ◆◇ 15% (*w/v*), ▲△ 20% (*w/v*); (**a**) glucose; (**b**) xylose; (**c**) arabinose; (**d**) lactic acid. Compounds concentrations (g/L) (filled symbol) and yield (open symbol), expressed as g compound per 100 g of compound in BSG. Experiments were conducted in duplicate. Analytical measurements were conducted in triplicate.

Untreated BSG was submitted to enzymatic hydrolysis by using a commercial cellulolytic complex (Celluclast 1.5 L). Different solids’ loading concentrations (2, 5, 10, and 20% *w/v*) were assayed. As can be observed in Figure 3a, the maximum glucose concentration was detected after 22 h for all the solids’ concentrations evaluated in this work. The concentration of glucose in the hydrolysates drastically decreased after this maximum due to the formation of lactic acid during this period (see Figure 3d). Trigueros et al. [25] also found high concentrations of lactic acid in enzymatic hydrolysates obtained with cellulases from the industrial solid residue of red seaweed after agar extraction. According to the literature, lactic acid fermentation may occur spontaneously when anaerobic conditions, water activity, moisture, salt concentration, and temperature are favorable for the growth of the autochthonous lactic acid bacteria [26]. 

The maximum concentration of glucose increased from 1.88 ± 0.07 g/L to 7.24 ± 0.01 g/L by increasing the substrate concentration from 5% to 20%, corresponding to hydrolysis yields of 18.9 ± 0.7% and 18.2 ± 0.2%, respectively. No substantial differences were found in the hydrolysis yield when solids concentration was increased, although the highest yield (23.0 ± 0.4%) was reached for 10% of solids’ loading. The glucose concentration obtained in these hydrolysates was too low to be submitted to further fermentation to obtain bioethanol in a feasible way. Similar results can be found in the literature for untreated BSG with different cellulases. For instance, Michelin and Teixeira [27] achieved a cellulose conversion to glucose of 36.97 ± 1.76% when performing enzymatic hydrolysis of 5% (*w*/*v*) untreated BSG for 72 h with Cellic Ctec2 and NS 22083 (Novozymes). Alonso-Riaño et al. [28] reported a glucose yield of about 40% from untreated craft BSG by using the cellulase 1,4-(1,3:1,4)-β-d-Glucan 4-glucanohydrolase, EC 3.2.1.4, from *Aspergillus niger* (Sigma-Aldrich, St. Louis, MO, USA).

#### 3.2.2. Subcritical Water-Pretreated BSG

**Figure 4:**
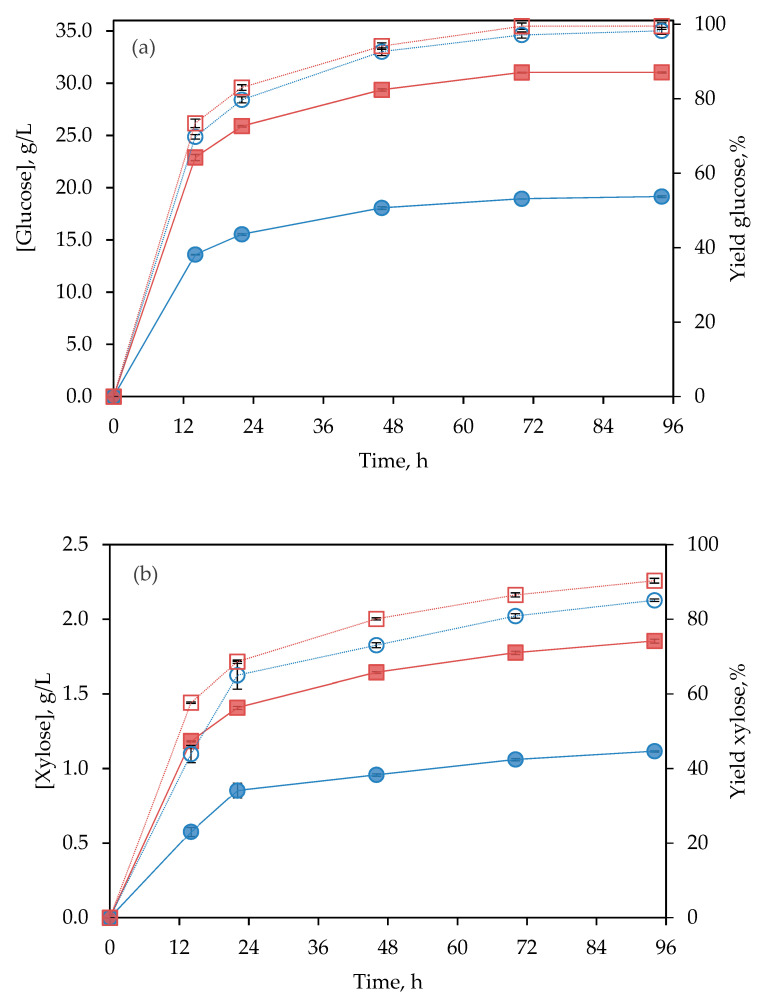
Time course of enzymatic hydrolysis of subW-pretreated BSG at different solids’ loading ●○ 5% (*w/v*), ■□ 8% (*w/v*); (**a**) glucose; (**b**) xylose. Monosaccharides’ concentration (g/L) (filled symbol) and yield (open symbol), expressed as g monosaccharide per 100 g of monosaccharide in pretreated BSG. Experiments were conducted in duplicate. Analytical measurements were conducted in triplicate.

To improve enzymatic digestibility, BSG was submitted to subW. Almost a complete conversion of cellulose to glucose has been achieved for both solid concentrations assayed, 98.2% and 99.5% for 5% and 8% (*w/v*) solids, respectively. That means a glucose concentration of 19.15 ± 0.09 and 31.05 ± 0.06 g/L, respectively, after 94 h of treatment (see Figure 4a). Nevertheless, after 46 h of treatment, the increase in glucose concentration in the hydrolysates becomes too slow to be convenient in terms of productivity, reaching at this time over 94% of the maximum glucose concentration achieved during the entire process for both assays. Lactic acid was not found in the enzymatic hydrolysates from subW-pretreated BSG, suggesting that this pretreatment could also act as a sterilizing treatment, preventing the development of lactic acid bacteria and avoiding the step of autoclaving, reducing the costs of bioprocesses. 

Enzymatic hydrolysis assays were not carried out at higher solid concentrations with subW-pretreated BSG, as the obtained slurry was a semi-solid pulp so hygroscopic that it was impossible to promote a good mass contact between enzymes and the polysaccharides. In this case, the buffer solution was much more retained by the pretreated solids, according to the higher WRV of subW-pretreated BSG compared to untreated BSG. 

To improve enzymatic hydrolysis, different pretreatments have been studied in order to make BSG cellulose more accessible to enzymes. Mussatto et al. [29] performed a two-step pretreatment with sulfuric acid and sodium hydroxide to obtain a hydrolysate with 59 g/L of glucose (73.8% yield) from 8% (*w/v*) of pretreated BSG by using Celuclast 1.5 L for 92 h. Rojas-Chamorro et al. [30] reached a cellulose–glucose conversion of 70% after pretreatment of BSG at 160 °C with 6.83% H_3_PO_4_ (*w/v*) and further enzymatic hydrolysis with Cellic CTec3 (Novozymes A/S, Bagsværd, Denmark) supplemented by fungal β-glucosidase (Novozyme 50010), during 48 h at 5% (*w/v*). These previous studies reported a lower hydrolysis yield than this work. However, Rojas-Chamorro et al. [14] also achieved a high enzymatic digestibility from 97.4 to 99.7% from BSG pretreated in an aqueous phosphoric acid solution 2% (*w/v*) at 155 °C, with solids’ loading of 5% and 15% (*w/v*), respectively.

Comparing the different results for cellulase treatment of raw BSG, and subW-pretreated BSG at 5% of solids loading, it can be observed that the maximum concentration of glucose reached, in each hydrolysate, was more than 10-fold higher for pretreated BSG than for raw BSG. As shown in Table 1, subW-pretreated BSG showed a 1.8-fold higher glucans content than untreated BSG. Furthermore, the glucose yield for subW-pretreated BSG was 5.2-fold higher than for raw BSG. During the pretreatment, the removal of the hemicelluloses’ fraction favors enzymatic hydrolysis, making cellulose chains more accessible to enzymatic attack, for conversion into glucose monomers [8,9,10]. In this work, the cellulose/hemicelluloses ratio in pretreated BSG was more than 34-folder higher than in raw BSG. Additionally, xylose, xylo-oligomers, and xylan are strong inhibitors of cellulose hydrolysis by enzymes [31], thus reducing the xylan content during the subW-pretreatment is also convenient. On the other hand, the lignin accumulation on subW-pretreated BSG did not reduce the enzymatic yield.

Time course for pentoses for untreated and subW-pretreated BSG have been plotted in Figure 3b,c, and Figure 4b. Lower xylose yields were obtained for untreated BSG than for subW-pretreated BSG, whereas arabinose was not detected in enzymatic hydrolysates from pretreated BSG, consistently with its complete release during subW treatment, as discussed above. Therefore, Celluclast 1.5 L was able to release not only glucose but also pentoses from both untreated and pretreated BSG. This observation agrees with previous reports, which indicated that Celluclast 1.5 L demonstrated hemicelluloses-degrading activity [32,33]. 

### 3.3. Separate Hydrolysis and Fermentation (SHF)

**Figure 5:**
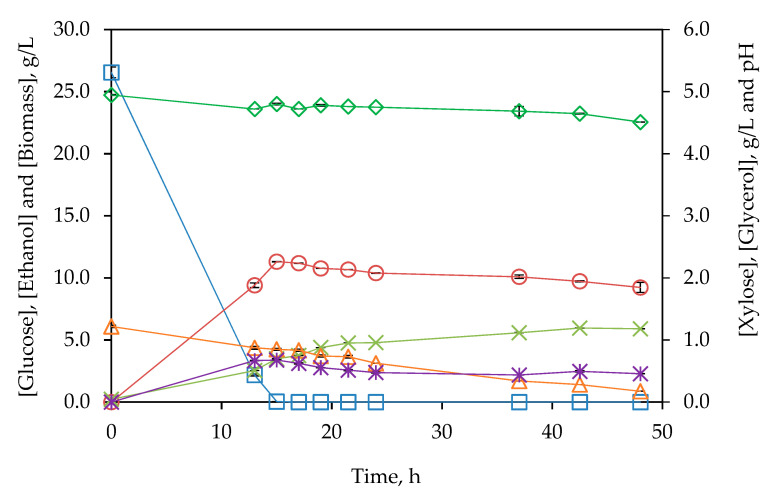
Evolution of ◇ pH and □ glucose, △ xylose, ○ ethanol, ∗ glycerol and x biomass concentrations (g/L) for fermentation from subW-pretreated BSG after enzymatic hydrolysis (48 h and 8% *w/v*) in a SHF configuration during 48 h. Analytical measurements were conducted in triplicate.

The hydrolysate obtained from subW-pretreated BSG by enzymatic hydrolysis with 8% (*w*/*v*) of solids for 48 h at 50 °C was autoclaved at 120 °C for 20 min and then submitted to fermentation by Ethanol Red^®^. After autoclaving, the hydrolysate composition was 31.13 g/L glucose and 1.43 g/L xylose. The fermentation media contained 85% of this hydrolysate. Figure 5 shows profiles of pH and biomass, glucose, xylose, ethanol, and glycerol concentration.

At the beginning of the fermentation assay, the sugars’ concentrations were 26.55 g/L glucose and 1.22 g/L xylose. After 13 h of fermentation, 92% of glucose was consumed, with a glucose depletion at 15 h. At this time, the greatest level of ethanol (11.30 ± 0.01 g/L) was produced. A slight decrease in ethanol concentration was observed after reaching its maximum, together with an increase in biomass concentration. Branco et al. [34] already observed a slight increment in biomass concentration when ethanol started to be reassimilated due to the ability of *S. cerevisiae* to consume ethanol when fermentable sugars dropped. In this regard, biomass concentration still increased after glucose exhaustion also due to consumption of xylose and probably other metabolites present in the hydrolysate. After 48 h of fermentation, the biomass increased from 0.228 ± 0.007 to 5.901 ± 0.005 g/L, corresponding to a biomass/substrate yield of 0.206 ± 0.003 g/g.

As expectable, xylose contribution to ethanol production was not remarkable since pentose sugars are not easily metabolized. Amândio et al. [35] also reported slight xylose consumption by Ethanol Red^®^. In the present work, the maximum concentration of glycerol, 0.677 ± 0.005 g/L, was achieved at 13 h, showing a minor decrease after that. The reduced glycerol production indicates that most of the glucose was fermented to ethanol. 

Some authors have been evaluating different pretreatments for bioethanol production with *S. cerevisiae* from BSG. Table 2 summarizes the operation conditions and the results reported for pretreatment, enzymatic hydrolysis and fermentation assays from BSG in these studies, together with some results obtained in this work. The ethanol yield from this study at 8% (*w/v*) in SHF configuration was 83%. This result was consistent with the reported data collected in Table 2, ranging from 55 to 81%, obtained by using acid or alkali pretreatments. Chen et al. [10] reported a fermentation yield of 90% from subW-pretreated wheat straw at 15% (*w/v*) solids loading in SHF configuration. This preliminary finding suggests the potential of subcritical water as a pretreatment of lignocellulosic material for bioethanol production.

### 3.4. Simultaneous Saccharification and Fermentation (SSF)

#### 3.4.1. Batch Strategy

**Figure 6:**
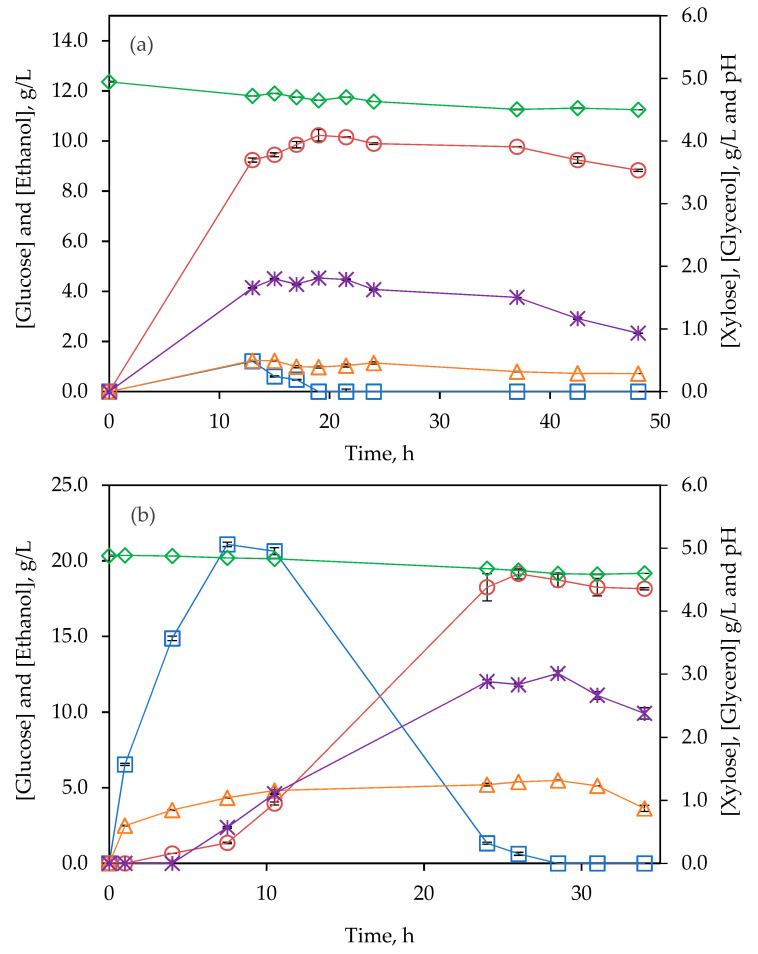
Evolution of ◇ pH and □ glucose, △ xylose, ○ ethanol and ∗ glycerol concentrations for fermentation from subW-pretreated BSG (**a**) 8% (*w/v*) and (**b**) 15% (*w/v*) in a SSF configuration. Analytical measurements were conducted in triplicate.

subW-pretreated BSG was subjected to a SSF process at 8%(*w/v*) solids loading, the same tested in the previous SHF. Figure 6 shows profiles of pH, glucose, xylose, ethanol, and glycerol concentration along 48 h of SSF. After 13 h of fermentation, glucose concentration was still 1.21 g/L, while ethanol concentration was 9.24 g/L, increasing slowly until reaching the highest value of 10.23 g/L at 19 h. The glucose concentration needed to produce this ethanol concentration increase (0.99 g/L) is 1.94 g/L, whereas the glucose concentration detected decreased 1.21 g/L. Furthermore, a reduced amount of glycerol was formed during this period, 0.16 g/L, indicating that glucose has still been released from cellulose during the period between 13 and 19 h.

Table 3 summarizes and compares the maximum ethanol concentration, yield (as a percentage of the theoretical), yield_ethanol/solid_ and volumetric productivity obtained in the SHF and SSF assays carried out in this work. The ethanol yield was calculated according to Equation 7 for SHF and SSF to compare the values on the same basis.

The ethanol content of 10.2 ± 0.2 g/L was obtained after SSF fermentation at 8% (*w/v*) solids loading, compared to 11.30 ± 0.01 g/L for SHF mode, corresponding to a yield of 64.2 ± 0.8 and 83.4 ± 0.4%, respectively. Similarly, Rojas-Chamorro et al. [14] reported a higher value of ethanol yield for SHF mode (72% after 72 h enzymatic hydrolysis + 24 h fermentation) as compared to SSF (62% after 72 h). Glucose accumulation was not observed during the assay, which may indicate a poor conversion of cellulose into glucose by working at 38 °C, since cellulases are more efficient at 50 °C. Moreover, the ratio ethanol/glycerol produced by SHF was 17, compared to 6 by fermentation in SSF mode. According to the literature, an increase in glycerol formation led to a decrease in ethanol production [40]. Although the ethanol yield was lower using the SSF mode instead of SHF configuration, the time to reach the maximum ethanol concentration was reduced from 63 h in SHF to 19 h in SSF. In this regard, the volumetric fermentation productivity in the SHF and SSF processes, at 8% of solids loading, were 0.1794 ± 0.0002 and 0.54 ± 0.01 g·L^−1^·h^−1^, respectively (see Table 3). Therefore, from an economic point of view, the use of SSF instead of SHF may be advantageous by reducing the total time required and also because only one vessel/reactor is need.

Even that, the ethanol concentrations reached by both configurations, SHF and SSF, at 8% solids loading were not high enough to be considered for a second-generation bioethanol process, being necessary at, at least, 4% by volume for distillation economic feasibility [41]. For this reason, SSF assays with 15% solids loading were performed (see Table 3). Similar ethanol yields were reached at 8% and 15% solids loading, 64.2% and 64.0%, respectively. However, an ethanol concentration of 19.1 g/L was reached at 15% solids loading after 26 h (see Figure 6b). Although the time needed to achieve the maximum level of ethanol was higher at 15% than at 8%, 26 h vs. 19 h (see Figure 6), the volumetric ethanol productivity was higher at 15%, reaching 0.74 instead of 0.54 g·L^−1^·h^−1^.

SSF was also attempted with 25% of solids loading; however, the hygroscopic behavior of the solid was so high, according to the WRV of the subW-pretreated BSG presented in Section 3.1, that no liquefaction of the slurry was achieved, even after 96 h. It was impossible to perform the hydrolysis preventing the fermentation. In order to overcome this limitation, a fed-batch strategy was followed to achieve a total solids’ loading of 25% (*w/v*).

#### 3.4.2. Fed-Batch Strategy

**Figure 7:**
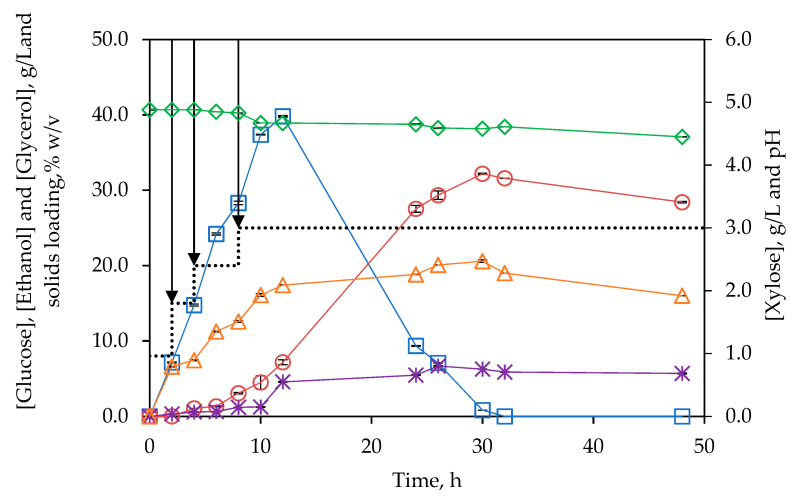
Evolution of ◇ pH and □ glucose, △ xylose, ○ ethanol and ∗ glycerol concentrations for fermentation from subW-pretreated BSG at a total solids’ loading of 25% (*w/v*) following a fed-batch SSF configuration. The discontinuous line shows solids’ charges over time. Analytical measurements were conducted in triplicate.

Figure 7 shows the evolution of pH and glucose, xylose, ethanol, and glycerol concentrations for the SSF assay performed at 25% solids loading following a fed-batch strategy. The assay was initiated with a solid concentration of 8%, and subW-pretreated BSG was added after 2, 4, and 8 h of fermentation (see Section 2.6.2), achieving a total solids loading of 25% (*w/v*).

Compared with SSF at 8 and 15% solids loading, a similar yield was reached, 64.7%, whereas a maximum level of ethanol, 32.18 g/L was produced after 30 h. This value corresponds to 4.08% (*v/v*), achieving the threshold of 4%, as discussed above. The same ethanol/glycerol ratio of 6 was found in all the SSF assays performed in this work. 

The value of ethanol concentration from BSG obtained in this study was higher than that reported in earlier works using acid and/or alkali-pretreated BSG, ranging from 12.8 to 22.7 g/L, as collected in Table 2, while being lower than that obtained by recent research on BSG (42.3 g/L and 39.0 g/L) using the whole slurry of pretreated BSG through different co-fermentation strategies [42,43]. Pinheiro et al. [43] achieved ethanol concentrations from 28.7 to 42.3 g/L by fermentation of the whole slurry, obtained by hydrolysis of BSG at 160 °C and 25% solids loading, after 120 h of saccharification. In this case, the productivity was lower than 0.352 g·L^−1^·h^−1^, considering only the saccharification time as the fermentation time was not reported. According to the authors, the ethanol concentration varied depending on the chemical composition of the raw BSG tested. In this regard, the maximum ethanol level was achieved from BSG with a total glucan content of 32.1% (7.6% of soluble glucose polysaccharides) compared with 19.1% glucan content in the BSG used in this work. Otherwise, Rojas-Chamorro et al. [42] reached the concentration of 39 g/L by following a co-fermentation strategy with *Escherichia coli* from BSG pretreated with phosphoric acid after 24 h of saccharification and 100 h of fermentation, which means a productivity of 0.315 g·L^−1^·h^−1^. Furthermore, these authors introduced an intermediate step of concentration of the enzymatic hydrolysate by evaporation. In both works, the productivity was noticeably lower than that achieved in this work by fed-batch SSF, 1.073 g·L^−1^·h^−1^, as the total time required for ethanol production from BSG at its maximum level has been reduced to 30 h. Likewise, according to the author’s knowledge, the overall ethanol productivity obtained in this study is the highest reported to produce bioethanol from BSG and eventually it could be increased by adding more solids charges, using more than 25% of solids’ loading.

### 3.5. Process Overview

**Figure 8:**
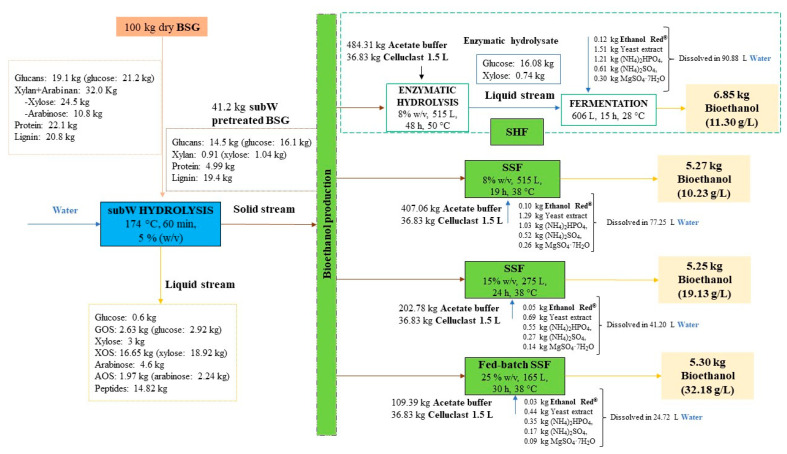
Diagram flow for bioethanol production from subW-pretreated BSG by saccharification and fermentation at different operational modes. “GOS”,” XOS”, and “AOS” stands for gluco-oligomers, xylo-oligomers, and arabino-oligomers, respectively. Calculation has been conducted considering no solvent retention.

Figure 8 shows a diagram flow for bioethanol production from BSG under the different conditions investigated in this work, pretreatment with subcritical water at 174 °C for 60 min and 5% (*w/v*), enzymatic hydrolysis of the pretreated biomass at 8% (*w/v*) solids loading and subsequent fermentation, as well as simultaneous saccharification and fermentation (SSF) with different solids’ concentrations. Process overview is presented considering 100 kg of dry BSG as a calculation basis. The subW liquid stream contained 32.3 kg of carbohydrates (3.51 kg glucose, 21.9 kg xylose and 6.85 kg arabinose) mainly in oligomeric form and 14.82 kg peptides. Different options could be followed to valorize the subW liquid stream. For instance, after purification, xylo-oligomers and peptides may be used as a functional food ingredient [44]. On the other hand, the subW liquid stream could be submitted to fermentation after converting the oligomer sugars into monomers, as monomers are present at low concentrations (0.30 g/L glucose, 1.5 g/L xylose and 2.3 g/L arabinose). Furthermore, it could be used to produce other valuable building blocks as xylose, which can be converted to xylitol or furfural [45,46]. 

The subW pretreatment yielded 41.2 kg of pretreated solid, which contained 16.1 kg glucose, 1.04 kg xylose, 4.99 kg protein, and 19.4 kg lignin. The subW-pretreated solid was submitted to enzymatic hydrolysis at 8% (*w/v*) solids loading releasing 16.08 kg glucose and 0.74 kg xylose. Table 4 collects the mass balance obtained for glucose, xylose, arabinose, proteins, and lignin of subW hydrolysis at 5% (*w*/*v*) following to enzymatic hydrolysis at 8% (*w/v*) solids loading, considering an initial amount of 100 kg of dry BSG. The mass of each compound in the solid streams has been calculated from the chemical composition of raw BSG (m_feed_), subW-pretreated BSG (m_subW-pretreated-BSG_) and solid residue remained after enzymatic hydrolysis of subW-pretreated BSG at 8% (*w/v*) solids loading (m_E-PBSG_) collected in Table 1, considering the mass of solid residues after each treatment. The mass of each compound in the liquid streams has been evaluated from the concentration of each compound in the subW hydrolysate, m_subW-hydrolysate_, (see Table 1) and that in the enzymatic hydrolysate (m_enzymatic-hydrolysate_) and the solids’ loading in each treatment, 5% and 8% (*w/v*), respectively. The outlet mass (m_outlet_) and the mass balance (MB) for each compound was calculated according to Equations (11) and (12), respectively:

(11) $$m_{\mathrm{outlet}}\;\left(\mathrm{kg}\right)=m_{\mathrm{subW}-\mathrm{hydrolysate}\;}\left(\mathrm{kg}\right)+m_{\mathrm{enzymatic}-\mathrm{hydrolysate}}\left(\mathrm{kg}\right)+m_{E-\mathrm{PBSG}}\left(\mathrm{kg}\right)$$

(12) $$\mathrm{Mass}\;\mathrm{balance},\mathrm{MB}\;\left(\%\right)=\frac{m_{\mathrm{compound},\mathrm{outlet}}\;\left(\mathrm{kg}\right)}{m_{\mathrm{compound},\;\mathrm{feed}}\;\left(\mathrm{kg}\right)}\cdot 10$$

The overall recovery of carbohydrates after pretreatment and enzymatic hydrolysis was 87%, with glucose and xylose recoveries higher than 90% (see Table 4). Arabinose recovery was lower (64%) due to the formation of degradation products such as furfural [47]. Most of the carbohydrates originally presented in untreated BSG has been solubilized and recovered during the combined process (57% by subW hydrolysis and 30% by enzymatic hydrolysis), with only 0.6% remaining in the solid residue (E-PBSG). The solid remained after enzymatic hydrolysis at 8% (*w*/*v*) solids contained 87.6% of lignin (see Table 1), which can maximize the utilization of this lignocellulosic by-product. The production of lignin-derived biochemicals, such as catechol, cresol, phenol, and acetic acid under a biorefinery approach considerably reduces the minimum price selling of bioethanol [48].

In addition, the HHV of this solid residue was calculated from the elemental composition collected in Table 5. The HHV and elemental composition of raw BSG, subW pretreated BSG and the solid residue remaining after SSF at 25% solids loading are also presented in Table 5. The HHV of both raw BSG and subW-pretreated BSG are about 20 MJ/Kg, whereas the HHV of the solids remained after enzymatic hydrolysis, and the SSF increased up to more than 30 MJ/kg. The latest value is similar to that of ethanol 100% (29.7 MJ/kg) and petroleum coke (31.3 MJ/kg) [49]. The high HHV of the residual solid also addresses the opportunity to produce energy by combustion that can be employed within the process.

Depending on how the fermentation processes were conducted, the energy and chemical requirements of the process and the amount of ethanol produced varied. For instance, 6.85 kg of ethanol were obtained from 100 kg of dry raw BSG when SHF was performed, while around 5.3 kg were produced by SSF. However, to determine which process configuration is most interesting for the industrial production of ethanol from BSG, it would be necessary to carry out a techno-economic evaluation. Consequently, it should be noted that the total volume per 100 kg of untreated BSG needed for SSF at 25% (*w/v*) solids loading is 3.8-folder lower than for SHF at 8% solids loading, and the ethanol concentration is 2.8-folder higher by performing SSF at 25% (*w*/*v*) solids loading. This fact not only means lower water, supplementation and yeast requirements and lower equipment costs but also a reduction in distillation costs. Furthermore, the productivity of the overall process was 6-fold higher in SSF with 25% (*w/v*) solids loading.

## 4. Conclusions

The bioconversion of brewer’s spent grain using subcritical water as pretreatment, followed by simultaneous saccharification and fermentation by Ethanol Red^®^ yeast is a promising option to achieve the integral valorization of this by-product. The subW pretreatment at 174 °C for 60 min resulted in an easily hydrolysable solid with higher glucan content than raw BSG, yielding a glucose release of 99.5%. Separated hydrolysis and fermentation (SHF) and simultaneous saccharification and fermentation (SSF) of pretreated BSG, at different solids’ loading, were evaluated in terms of ethanol production. Fed-batch SSF allowed one to increase the total solids’ loading to 25% (*w/v*), resulting in a relatively high ethanol concentration (>32 g/L) in only 30 h, achieving the target concentration of 4% by volume. Furthermore, the highest value of ethanol productivity (1.07 g·L^−1^·h^−1^) reported for this raw material was attained. Therefore, second-generation bioethanol was obtained from lignocellulosic biomass, at a concentration above the 4% (*v/v*) of ethanol required for an economically feasible distillation, by using green technologies such as subcritical water pretreatment avoiding chemicals’ addition and enzymatic hydrolysis.

## Figures and Tables

**Table 1:** Chemical composition of raw BSG, subW-pretreated BSG, solid residue remained after enzymatic hydrolysis of subW-pretreated BSG at 8% (*w/v*) solids loading (E-PBSG) and concentration of each compound in the subW hydrolysate expressed as g of compound per 100 g of dry raw BSG. Characterization determinations were conducted in duplicate. Analytical measurements were conducted in triplicate. Values are expressed as the mean ± standard deviation from replicate determinations.

**Compound**	**Raw BSG** **(% dwb)**	**subW-Pretreated BSG (% dwb)**	**subW Hydrolysate (g/100 g_BSG_)**	**E-PBSG** **(% dwb)**
Glucans	19.1 ± 0.2	35.1 ± 0.3	3.16 ± 0.02	1.00 ± 0.01
Starch	4.11 ± 0.06	-	-	-
Β-glucan	0.99 ± 0.01	-	-	-
Cellulose	14.0 ± 0.2	-	-	-
Hemicelluloses	32.0 ± 0.6	2.22 ± 0.04		0.150± 0.001
Xylan	21.6 ± 0.4	2.22 ± 0.04	19.28 ± 0.09	0.150 ± 0.001
Arabinan	9.5 ± 0.4	Not detected	6.03 ± 0.01	Not detected
Acetate	0.93 ± 0.05	Not detected	-	Not detected
Lignin	20.8 ± 0.2	47.1 ± 0.5	-	87.60 ± 0.05
Acid insoluble	15.5 ± 0.1	42.9 ± 0.4	-	85.78 ± 0.04
Acid soluble	5.3 ± 0.2	4.2 ± 0.1	-	1.82 ± 0.03
Ash	3.32 ± 0.06	2.7 ± 0.1	-	3.1 ± 0.1
Proteins	22.1 ± 0.7	12.1 ± 0.1	14.82 ± 0.04	19.0 ± 0.9

**Table 2:** Comparison between the results obtained for SHF (8% *w/v*) and fed-batch SSF (25% *w/v*) assays from pretreated BSG with *S. cerevisiae* in this work and in the literature.

Pretreatment	Enzyme.	Enzyme Dosage	Conf	Solid Loading (% *w/v*)	[Glucose] (g/L)	Glucose Yield (%)	[Ethanol] ^(1)^ g/L	Ethanol Yield (%)	Productivity ^(2)^ (g·L^−1^·h^−1^)	Reference
SubW, 174 °C, 50 bar, 60 min.	Celluclast 1.5L	40 FPU/g_BSG_.	SHF	8	31.1	99.7	11.3	^(1)^ 83	0.75	This work
			Fed-batch SSF	25	-	-	32.2	65	1.07	
H_3_PO_4_ (6.83%), 160 °C	Cellic CTec3 β-glucosidase	15 FPU/g_substrate_ 15 IU/g_substrate_	SHF	15	59.4		22.6	72	0.94	[14]
			SSF		-	-	18.5	62	0.60	
H_2_SO_4_ (1%), 130 °C, 26 min	Cellic CTec3 β-glucosidase	15 FPU/g_substrate_ 15 IU/g_substrate_	SSF	25			27	68		[36]
1. H_2_SO_4_ (1.25%), 120 °C, 17 min 2.NaOH (2%), 120 °C, 90 min	Cellulase β-glucosidase (Novozymes)	2.24% (*v/v*) 1%(*v/v*)	SHF	8	75	97	12.8	54.8	0.53	[37]
NaOH (5%), 50 °C, 12 h	Cellic^®^CTec2	51 FPU/g_solid_ (calculated)	SHF	15	41.7		17.3	81	N.R.	[38]
1. H_2_SO_4_ (1.8 5%), 90 °C, 19.5 min 2. H_2_SO_4_ (1%), 120 °C, 30 min	Cellic^®^ CTec3	0.04 g/g_dry-matter_	SHF	15	46.1	75.5	16.9	71.6	0.72	[39]

“Conf” stands for configuration. “NR” stands for not reported. ^(1)^ Ethanol yield calculated according to Equation (6). ^(2)^ Productivity refers to volumetric ethanol productivity of fermentation step.

**Table 3:** Parameters obtained in SHF and SSF of subW-pretreated BSG at different solids’ loadings with Ethanol Red^®^. Analytical measurements were conducted in triplicate. Values are expressed as the mean ± standard deviation from triplicate determinations.

Configuration	Solids Loading (%)	[Ethanol]_max_ (g/L)	Ethanol Yield (%)	Y_ethanol/solid_ (g/g)	Productivity (g·L^−1^·h^−1^)
SHF	8	11.30 ± 0.01 ^b^	83.4 ± 0.4 ^b^	0.1662 ± 0.0002 ^b^	^(1)^ 0.754 ± 0.001 ^d^ ^(2)^ 0.1794 ± 0.0002 ^a^
SSF	8	10.2 ± 0.2 ^a^	64.2 ± 0.8 ^a^	0.128 ± 0.003 ^a^	0.54 ± 0.01 ^b^
SSF	15	19.1 ± 0.3 ^c^	64.0 ± 0.6 ^a^	0.128 ± 0.002 ^a^	0.74 ±0.01 ^c^
Fed-batch SSF	25 (8 + 7 + 5 + 5)	32.18 ± 0.08 ^d^	64.7 ± 0.3 ^a^	0.1287 ± 0.0003 ^a^	1.073 ±0.003 ^e^

^(1)^ Productivity considering the time of the fermentation step (15 h). ^(2)^ Productivity considering the time of saccharification and fermentation (48 + 15 h). Values are expressed as mean ± standard deviation from triplicate determination. Values with different letters in each column are significantly different when applying the Fisher’s least significant differences (LSD) method at *p*-value ≤ 0.05.

**Table 4:** Mass balance (MB) of subW hydrolysis at 5% (*w*/*v*) and subsequent enzymatic hydrolysis at 8% (*w/v*) solids loading from 100 kg of dry BSG. Mass of each compound in feed (m_feed_), subcritical water hydrolysate (m_subW-hydrolysate_), subW-pretreated BSG (m_subW-pretreated-BSG_), enzymatic hydrolysate (m_enzymatic-hydrolysate_), solid residue remained after enzymatic hydrolysis of subW-pretreated BSG at 8% (*w*/*v*) solids loading (m_E-PBSG_) and outlet mass (m_outlet_) calculated as the sum of each compound in the different final streams (m_subW-hydrolysate_ + m_enzymatic-hydrolysate_+ m_E-PBSG_).

Compound	m_feed_ (kg)	m_subW-hydrolysate_ (kg)	m_subW-pretreated-BSG_ (kg)	m_enzymatic-hydrolysate_ (kg)	m_E-PBSG_ (kg)	m_outlet_ (kg)	MB (%)
Solid	100	-	41.2	-	24.5	-	-
Glucose	21.2 ± 0.2	3.51 ± 0.02	16.1 ± 0.1	16.08 ± 0.26	0.27 ± 0.00	19.9 ± 0.3	94 ± 2
Xylose	24.5 ± 0.5	21.9 ± 0.1	1.04 ± 0.02	0.74 ± 0.01	0.04 ± 0.00	22.7 ± 0.1	92 ± 2
Arabinose	10.8 ± 0.5	6.85 ± 0.01	Not detected	Not detected	Not detected	6.85 ± 0.01	64 ± 3
Carbohydrates	56.5 ± 0.7	32.3 ± 0.1	17.1 ± 0.1	16.8 ± 0.3	0.31 ± 0.00	49.4 ± 0.3	87 ± 2
Protein	22.1 ± 0.7	14.85 ± 0.04	4.99 ± 0.06	Not determined	4.8 ± 0.2	19.6 ± 0.2	89 ± 4
Lignin	20.8 ± 0.2	Not determined	19.4 ± 0.2	Not determined	21.42 ± 0.01	21.42 ± 0.01	103 ± 1

Glucose, xylose and arabinose present as glucan, xylan, and arabinan, respectively, have been converted to glucose, xylose, and arabinose by applying the corresponding anhydrous factor. Calculation was conducted considering no solvent retention.

**Table 5:** (CHN) and estimated heating value (HHV) of raw BSG, subW-pretreated BSG, solid residue remaining after enzymatic hydrolysis of pretreated BSG at 8% (*w/v*) solids loading (E-PBSG) and solid residue remaining after SSF attempt of pretreated BSG at 25% solids loading (SSF-PBSG). Values are expressed as mean ± standard deviation from triplicate determination.

	Raw BSG	subW-Pretreated BSG	E-PBSG	SSF-PBSG
C (% dwb)	49.1 ± 0.5	53.3 ± 0.7	65.5 ± 0.9	65.2 ± 0.5
H (% dwb)	6.64 ± 0.01	6.87 ± 0.07	8.45 ± 0.04	8.45 ± 0.06
N (% dwb)	3.5 ± 0.1	1.94 ± 0.02	3.0 ± 0.1	3.14 ± 0.01
HHV (MJ/kg)	20.1 ± 0.3 ^a^	22.0 ± 0.7 ^b^	31 ± 2 ^c^	30.3 ± 0.4 ^c^

HHV values with different letters in each column (a, b or c) are significantly different when applying the Fisher’s least significant differences (LSD) method (*p*-value ≤ 0.05).

## Data Availability

The data presented in this study are available on request from the corresponding author.
